# Evaluation of the Preoperative Antiseptic Efficacy of Ozone on Dog Skin in Comparison with Traditional Methods

**DOI:** 10.3390/vetsci12090843

**Published:** 2025-09-01

**Authors:** Pierre Melanie, Carlotta Niola, Federico Guerrini, Nicolò Pareto, Simone Mancini, Filippo Fratini

**Affiliations:** 1 Department of Veterinary Sciences, University of Pisa, Viale delle Piagge 2, 56124 Pisa, Italy; pierre.melanie@unipi.it (P.M.); simone.mancini@unipi.it (S.M.); filippo.fratini@unipi.it (F.F.); 2 DVM, Studio Veterinario Associato “Rossini-Silvestri”, Via Livornese 24, 56122 Pisa, Italy; 3 DVM, Ambulatorio Veterinario “De Guttry-Bertagna-Cassiodoro”, Via G.Amendola, 55042 Forte dei Marmi, Italy; fede.guerrini@icloud.com; 4 DVM, Studio Veterinario “Pontelungo”-Dott. Pareto, Via Brescia 41, 17031 Albenga, Italy; nico.pareto@gmail.com; 5 Interdepartmental Research Center “Nutraceuticals and Food for Health”, University of Pisa, Via del Borghetto 80, 56124 Pisa, Italy

**Keywords:** ozonated water, dogs, antiseptics

## Abstract

This paper aims to assess the antiseptic capacity of a double-distilled ozonated water solution and compare it to standard presurgical antiseptic protocols based on chlorhexidine and alcohol. Sixty-three canine patients undergoing different surgical procedures (such as thoracic, abdominal, proximal limb, or cervical) were enrolled in the study. Following extensive preoperative clipping of the selected anatomical regions, four standardized skin areas (6 × 6 cm each) were aseptically sampled according to a predefined protocol: Area 1 received soap-based chlorhexidine followed by alcoholic chlorhexidine; Area 2 was prepared using a combination of soap-based chlorhexidine and ozonated water; Areas 3 and 4 underwent presurgical skin antisepsis with a combination of ethyl alcohol and ozonated water, and ozonated water alone, respectively. Two different samples per area were collected using bacteriological buffers in order to evaluate the bacterial count at 90 s and 180 s, respectively. Those samples were sown via streak plating on Petri dishes using PCA (plate count agar) as the medium. The determination of the total mesophilic bacterial count on Petri dishes was used to assess the efficacy of the antiseptics in different areas. Results show that ozonated bidistilled water demonstrated comparable or superior efficacy compared to other antiseptics tested in this study. Its integration into surgical protocols could enhance patient safety while controlling environmental concerns associated with the massive use of chemical antiseptics.

## Simple Summary

Infections during surgery are a serious concern in both human and veterinary medicine. In order to minimize this risk, patients’ skin must be carefully cleaned and sterilized before any procedure. The objective of this study was to test the effectiveness of ozonated bidistilled water as an antimicrobial agent during preoperative surgical scrub in dogs. Researchers compared it with other commonly used antiseptics in veterinary practice, including alcoholic chlorhexidine, soap-based chlorhexidine, and ethyl alcohol. Sixty-three dogs undergoing abdominal or thoracic surgery were included in the study. The skin was divided into small areas, each treated with different antiseptics. The number of viable bacteria was measured before and after cleaning. The results show that ozonated water is as effective as traditional antiseptics.

## 1. Introduction

Skin in mammals represents a crucial protective barrier, separating the organism from the external environment. It performs vital functions such as protection against pathogens, regulation of body temperature, and sensory perception [1,2]. During surgical procedures, the skin barrier is compromised, exposing the animal to the risk of surgical site infections (SSIs), one of the major contemporary challenges in both human and veterinary medicine, given the increasing phenomenon of antibiotic resistance, the spread of hospital-acquired infections, and zoonotic risks [3,4]. These issues highlight the need for operating in an aseptic environment and adopting procedures aimed at antisepsis of the patient, as well as preparation and antisepsis of the surgeon, operating room, and instruments.

Both veterinary and human surgeons use various antiseptic agents during the cleaning and antisepsis phases prior to surgery; this preparation is also performed on the anatomical region that will be affected by surgery. In this study, two of the main and most common antiseptic agents—chlorhexidine and ethyl alcohol—are considered for comparison with ozone (O_3_), whose effectiveness and applications have been well known to the scientific community for years. The authors excluded the widely recognized antiseptic agent povidone–iodine due to an increasing preference for alternative antiseptic agents within local veterinary clinical protocols. Furthermore, povidone–iodine has been associated with reduced tolerability of the skin biofilm, frequently leading to irritation and allergic reactions [5,6].

Medical alcohol, in its forms of ethyl alcohol and isopropyl alcohol, is effective against a wide range of microorganisms, including both Gram-positive and Gram-negative bacteria, viruses, and fungi. It acts rapidly in its antimicrobial action, significantly reducing the bacterial load on the skin within seconds. The antimicrobial mechanism is well established, involving protein denaturation and disruption of lipid membranes, but it is believed that alcohol causes denaturation and precipitation of proteins, which in turn leads to damage to the cell membrane and eventual lysis [7,8]. Its application, typically recommended at high concentrations ranging from 60% to 80%, is straightforward and does not require rinsing, thus facilitating surgical field preparation.

Chlorhexidine is likely the most widely used biocidal agent in antiseptic products, including as a skin antiseptic, in formulations for oral mucosa, for antisepsis of the surgeon’s hands, and even as a preservative [8]. One of the main advantages of chlorhexidine, which makes it preferable even to the more widely known povidone–iodine solution, is its ability to adhere to the skin, releasing the active ingredient slowly, thus providing prolonged action over time and reducing the risk of postoperative infections [9,10]. Chlorhexidine is typically available in solutions ranging from 0.5% to 4% *w*/*v*, with the 2% *w*/*v* chlorhexidine gluconate (CHG) in a 70% *v*/*v* isopropyl alcohol (IPA) solution widely used, especially recommended for preoperative cleaning [11,12]. The mechanism of the antimicrobial activity of chlorhexidine involves positively charged molecules that bind to the negatively charged surface of bacterial cells, weakening the membrane integrity. This results in the loss of cytoplasm and precipitation of intracellular macromolecules, including proteins and nucleic acids, secondary to membrane disruption at low concentrations, while at higher concentrations, it causes membrane rupture [13,14].

In this context, there is growing interest in exploring alternative antiseptic methods, including ozone, which offers potential advantages in terms of efficacy, environmental impact, and patient safety. As a powerful oxidizing agent, ozone has demonstrated rapid microbial inactivation against a wide range of pathogens, including those resistant to other antiseptics [15]. This study was conceived as a preliminary investigation aimed at assessing the immediate quantitative reduction of bacterial load following antiseptic application, including ozonated water. It is not intended to replace or challenge existing preoperative protocols, but rather to provide baseline data to support future research and potential adjunctive use.

Ozone is distinguished by its capacity to induce a redox oxidative stimulus, which is crucial for intercellular and tissue signaling as well as the regulation of biological processes. Its antibacterial mechanism is primarily driven by its potent oxidative reactivity: ozone selectively targets the double bonds of unsaturated fatty acids within the bacterial cell membrane, initiating lipid peroxidation. This process disrupts the membrane’s integrity, altering its permeability and ultimately leading to cellular lysis. Additionally, ozone exerts oxidative effects on essential proteins and nucleic acids, thereby interfering with the bacterium’s metabolic and replicative functions. The formation of reactive oxygen species (ROS) and secondary peroxides further exacerbates intracellular oxidative damage, resulting in rapid and efficient microbial eradication without inducing the development of resistance [16,17].

These properties render ozone particularly advantageous in the treatment of infectious diseases. Its antibacterial efficacy stems from its ability to react with the double bonds of lipids in the bacterial membrane, thereby promoting oxidative damage to nucleic acids and amino acid side chains and generating biologically active compounds, such as the superoxide anion (O_2_^−^), hydroxyl radical (OH), and hydrogen peroxide (H_2_O_2_). These reactive species compromise the integrity of membrane phospholipids, ultimately leading to bacterial lysis. In Gram-positive bacteria, the primary target is the peptidoglycan layer of the cell wall, whereas in Gram-negative bacteria, the outer membrane is predominantly affected. Disruption of these structural components leads to extensive lysis and subsequent cellular inactivation [18].

Studies investigating infections caused by Gram-positive, Gram-negative, and MRSA bacteria exposed to ozone have demonstrated significant damage to the bacterial membrane [19,20], while prolonged exposure has been linked to mitochondrial alterations [21].

While the efficacy of commonly used antiseptic agents is well established and supported by extensive clinical validation, this study aims to offer a novel perspective by exploring the use of ozone as a skin antiseptic in preoperative procedures. Specifically, it investigates the potential of ozonated water as part of emerging antiseptic strategies, with efficacy potentially comparable to conventional protocols and distinct advantages, including greater biocompatibility and a favorable safety profile.

## 2. Materials and Methods

### 2.1. Inclusion Criteria

The study included sixty-three canine patients, regardless of breed, age, or sex, presenting for surgical procedures. The authors considered the weight and the available skin surface for sampling. Specifically, patients with a sampleable skin surface between 144 cm^2^ and 256 cm^2^ were included. The study areas involved the proximal regions of the limbs, as well as abdominal, thoracic, and cervical regions, ensuring an adequate surface area after patient trichotomy. Therefore, patients with a sampleable surface area of less than 144 cm^2^, or those undergoing dental procedures or surgical processes on the distal limb or tail areas with limited sampling surface, were excluded.

Additionally, subjects with previous contamination of the skin by alcohol, ultrasound gels, or other preoperative diagnostic products that could alter the normal skin microbiota were also excluded from the study.

### 2.2. Preparation of Ozonated Bidistilled Water and Other Antiseptic Supplies

The ozone generator (Herrmann Apparatebau GmbH^®^, Elsenfeld, Germany) was used to ozonize bidistilled water at room temperature to a concentration of 20 μg/mL via bubbling for ten minutes. The authors deliberately chose to extend the duration of bubbling to ensure optimal ozone saturation and to minimize potential fluctuations in concentration [19,22]. Although previous studies have indicated that ozone saturation in double-distilled water can be achieved within five minutes [23], the bubbling duration was extended in this study to mitigate potential concentration discrepancies.

In order to prolong the shelf life of the bidistilled water and enhance the stability of the ozone concentration, the solution was stored at 4 °C. It is noteworthy that the ozone has a reported half-life of approximately 10–30 min at room temperature in water, which can be extended to several hours at low temperatures and optimal pH [24].

A soap-based chlorhexidine solution was prepared in a glass container for the impregnation of non-sterile cotton gauzes. Additionally, gauzes saturated with Isopropyl alcohol and a solution of chlorhexidine in 95% alcohol (ESOFORM, Rovigo, Italy Cleansing solution for hands and skin 0.5%) were used for antiseptic purposes with a spray dispenser used for the precise application of the latter.

### 2.3. Sampling Procedure

**Figure 1:**
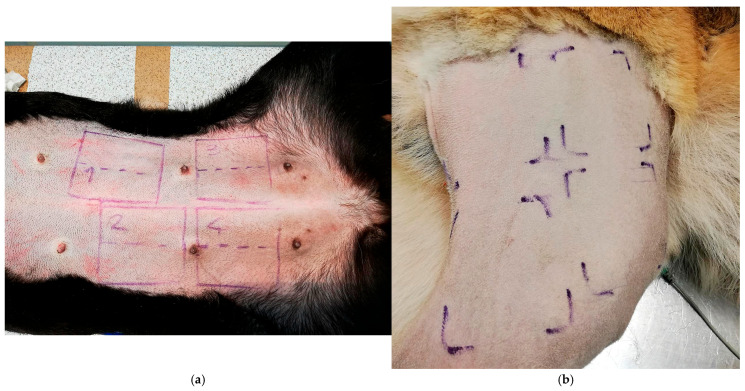
Graphical representation of the primary 6 × 6 cm sampling areas and their corresponding sub-zones (3 × 6 cm). The delineation of the sampling areas was performed using 6 × 6 cm sterilized metal templates and marked with a dermographic pen, thereby ensuring consistency and reproducibility of the sampling procedure across all subjects, including small-breed dogs (**a**). Preoperative clipping of the limb exposed a sufficient skin surface for standardized sampling (**b**).

Sterile Cultiplast^®^ (Milano, Italy) swabs were used for the sampling procedures according to their intended purpose: initial control, first sampling, and second sampling, each corresponding to the antiseptic agents applied. A GIMA^®^ (Gessate, Italy) dermographic pen and a 6 × 6 cm sterilized metal delimiter were used to precisely delineate the designated four primary sampling areas, as illustrated in Figure 1:

- Area 1: soap-based chlorhexidine + alcoholic chlorhexidine
- Area 2: soap-based chlorhexidine + ozonated water (20 µg/mL)
- Area 3: ethyl alcohol + ozonated water (20 µg/mL)
- Area 4: ozonated water (20 µg/mL)

The authors intentionally selected a 6 × 6 cm area as the standard sampling unit, as it represented the largest common surface area applicable to both large and small dogs within the inclusion criteria range (sampleable skin surface between 144 cm^2^ and 256 cm^2^).

Preoperative clipping in dogs undergoing procedures involving the cervical region or proximal limbs allowed for the exposure of a sufficiently wide skin surface, enabling the collection of four standardized 6 × 6 cm sampling areas even in subjects of varying body sizes. Although multiple anatomical sites were included, the study focused exclusively on the quantitative assessment of bacterial load reduction, without performing microbiological identification. This approach reflects common clinical practice, where antiseptic protocols are uniformly applied across different surgical sites, regardless of regional variations in resident microbiota, although such differences could theoretically influence antiseptic susceptibility and should be considered in future research.

An initial sterile swab was used to collect a representative sample of microorganisms from the previously shaved skin surface, serving as a “Control”. Subsequently, the authors subdivided the primary sampling non-prewashed areas into smaller zones measuring 3 × 6 cm, each corresponding to a specific antiseptic treatment and sampling time (90 s and 180 s denominates “P” and “S” series, respectively) to monitor any variations in microbial load. Furthermore, in Area 3, ethyl alcohol was combined with ozone to leverage its potent lipid-dissolving properties during mechanical friction, thereby enhancing the overall antimicrobial efficacy, which is conventionally augmented through association with other antiseptics [12].

The effectiveness of each antiseptic protocol was assessed on anatomically comparable but separate skin areas within the same subject, enabling a direct intra-individual comparison. This methodological approach reduced inter-subject variability and contributed to increasing uniformity and reliability of the collected data.

As an integral aspect of the comprehensive evaluation protocol, local irritation was assessed by direct visual inspection immediately prior to the surgical procedure and before any incision was made. The evaluation was based on predefined visual criteria for cutaneous inflammation, including erythema, swelling, and other visible changes.

The details of the zones and sampling times are summarized in the table below (Table 1).

All the bacteriological swabs used for sampling were immersed in 2 mL of sterile physiological solution and stored in the refrigerator at +4 °C, waiting to be transported in a refrigerated bag to the Microbiology Laboratory of the Department of Veterinary Sciences in Pisa.

### 2.4. Mesophilic Bacterial Count Determination

Samples were processed in a laminar flow hood to minimize the risk of contamination. For further microbiological analysis, 2 mL of sterile saline solution was added to each test tube and sown by streak plate method onto Petri dishes containing Agar Plate Count medium (Oxoid^®^, Milan, Italy). Plates were incubated at 30 °C for 72 h, followed by enumeration of viable mesophilic aerobic bacteria. In the absence of visible colonies, the respective result was noted as <10 CFU/cm^2^. The enumeration of colonies enabled the determination of the viable mesophilic bacterial load.

The total viable mesophilic bacterial count was expressed in log CFU/mL/cm^2^ (colony-forming units) according to a modified version of the ISO 7932 standard [25]. The following formula was applied:

$$log\;CFU/mL=\frac{\sum c}{V(n_{1}+0.1\times n_{2})\times d\times S}+40\%$$

where

- ∑c represents the total number of colonies counted for each dilution.
- *V* is the volume in millilitres (mL) of sterile saline solution in which the bacterial swab was immersed.
- $n_{1}\;$ denotes the number of dilutions performed.
- d indicates the dilution factor applied in the first dilution.
- S is the surface area analyzed, expressed in cm^2^.
- A correction factor of 40% was incorporated by the authors, grounded in scientific evidence suggesting that only approximately 60% of the microbial sample is recovered from the swab surface [26].

This methodology was employed to quantify the viable mesophilic bacterial load in the sampled areas, ensuring accurate and reproducible results in the context of the antiseptic efficacy evaluation.

### 2.5. Statistical Analysis

Log-transformed data (log_10_ CFU/cm^2^) were used to evaluate microbial load across experimental conditions. Nevertheless, due to the large number of results showing a bacterial load of zero—an expected outcome following surgical field disinfection procedures—it was not feasible to perform an ANOVA analysis; future studies could apply zero-inflated Poisson or negative binomial models to handle sparse microbiological CFU data. This limitation prevents the application of traditional statistical methods to a dataset that nonetheless carries clear scientific relevance. To underscore the significance of these findings, the authors chose to present them using Delta-log values expressed as log reduction (Δlog_10_ CFU/cm^2^). Pairwise comparisons between formulations were also performed between the P and S series, and Δlog values were used to quantify relative effectiveness.

Despite the promising results, this study has several limitations. The relatively small sample size and the high prevalence of zero bacterial counts limited the ability to perform robust statistical analyses. Future research with larger cohorts is necessary to confirm the findings and strengthen statistical power.

## 3. Results

All 63 patients were included in the final analysis, with no observed skin alterations or adverse effects, such as erythema, edema, or other local reactions, regardless of the assessment method.

**Figure 2:**
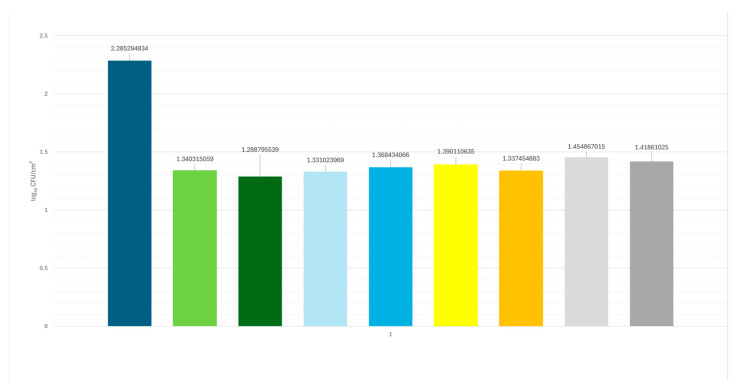
A graphical representation illustrates the equivalent efficacy of all the antimicrobial agents tested, highlighting the marked reduction in viable microbial counts for each treatment.

No statistically significant differences were observed among the antimicrobial agents used, either after the first treatment phase (P-series, 90 s) or after the second phase (S-series, 180 s). These findings confirm the antimicrobial effectiveness of all agents tested, as all treatment groups showed a reduction of approximately 1 log CFU/cm^2^ compared to the untreated control area (2.285 log CFU/cm^2^). In particular, the reduction ranged from 0.83 to 0.99 log units across all treatments, demonstrating comparable efficacy among the different antimicrobial combinations (Figure 2).

**Figure 3:**
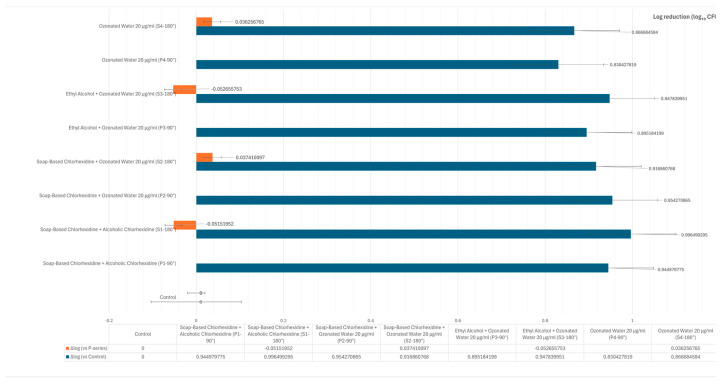
Illustration of Delta-log values expressed as log reduction compared to the untreated control (light blue bars). The effectiveness of each treatment is represented as the logarithmic reduction in microbial load (Δlog_10_ CFU/cm^2^) relative to the control (blue bars). The differences between the first (P-series) and second (S-series) treatment phases (orange bars) were minimal. Slightly lower efficacy was observed in the soap-based chlorhexidine + alcoholic chlorhexidine group (−0.051 log) and in the ethyl alcohol + ozonated water 20 µg/mL group (−0.052 log).

Regarding the statistical significance of the results between groups, no statistically significant differences were observed among the antimicrobial treatments at either the first treatment phase (P-series, 90 s) or the second treatment phase (S-series, 180 s). As shown in Figure 3, the reduction in bacterial load was consistent across all treatments. Specifically, the combination of soap-based and alcoholic chlorhexidine resulted in reductions of 0.944 log CFU/cm^2^ after 90 s and 0.996 log CFU/cm^2^ after 180 s. Comparable reductions were observed with the combination of soap-based chlorhexidine and ozonated water at 20 µg/mL (0.954 and 0.916 log CFU/cm^2^ at 90 and 180 s, respectively), and with ethyl alcohol combined with ozonated water (0.895 and 0.947 log CFU/cm^2^). Ozonated water alone also showed effective bacterial reduction (0.830 and 0.866 log CFU/cm^2^).

Despite these measurable reductions, the differences between groups did not reach statistical significance, indicating that all tested antimicrobial protocols demonstrated similar efficacy under the experimental conditions.

## 4. Discussion

The canine cutaneous microbiome is inherently composed of a complex and diverse population of aerobic, facultative anaerobic, and obligate anaerobic bacterial species [27]. In recent years, there has been increasing scientific interest in identifying antiseptic strategies that are not only effective in microbial load reduction but also environmentally sustainable and respectful of the host’s native microbial equilibrium. Within this framework, the present study was designed to comparatively evaluate the efficacy of various preoperative antisepsis protocols, with particular emphasis on the application of ozonated bidistilled water.

In the analysis of the results, the authors focused primarily on viable bacterial load, as it is a crucial determinant for assessing the effectiveness of the treatment and for monitoring the biological and clinical responses post-operatively. Due to the comparable efficacy of the various protocols, it is pertinent to critically examine the different antiseptic agents, emphasizing specific challenges associated with their use.

The findings indicate that ozone, when applied in the form of bidistilled water, achieves a reduction in bacterial load comparable to that obtained with chlorhexidine and ethyl alcohol, thereby supporting its potential role as a valuable adjunct in veterinary preoperative antiseptic protocols. This observation is particularly relevant in light of ozone’s advantageous characteristics, including its high biocompatibility, rapid onset of action, and low incidence of adverse effects when administered at controlled concentrations [28,29]. The antimicrobial and antiseptic properties of ozone are well documented in the scientific literature. Recent investigations have demonstrated its effectiveness both as a standalone agent and in synergistic combination with conventional antiseptics, highlighting its rapid bactericidal activity and minimal impact on skin barrier function in canine subjects [30]. Furthermore, other studies have emphasized ozone’s low cytotoxicity and broad-spectrum antimicrobial efficacy, including activity against multi-drug-resistant pathogens [17,19,31,32,33,34].

While ozone is distinguished by its rapid antimicrobial action and relatively low incidence of adverse effects, other antiseptics, such as chlorhexidine and alcohol, exhibit both advantages and limitations that influence their efficacy and safety in specific clinical contexts.

Ethyl alcohol is indeed effective in the presence of organic matter; however, it lacks sustained residual activity, meaning its antiseptic efficacy rapidly diminishes upon evaporation. Furthermore, while the effects are generally mild and localized, it may cause irritation and desiccation of the skin, which in turn increases the risk of cutaneous lesions, particularly in patients with heightened sensitivity [35]. Recent scholarly discourse indicates that, despite its practical utility, ethyl alcohol is often most effective when used in conjunction with other antiseptic agents, such as chlorhexidine, which can mitigate its limitations and enhance overall antimicrobial efficacy [12,36].

Consistent with its non-specific mechanism of action and the residual antibacterial effect, the use of chlorhexidine is widespread. However, there are several limitations, such as its potential toxicity to the eyes, ears, and brain tissue [37], and the risk of inactivation in the presence of spores or non-ionic surfactants, such as ethoxylated fatty alcohols and ethoxylated alkylphenols, which are commonly found in detergent products [8]. Additional drawbacks, as reported by the U.S. Food and Drug Administration (FDA), include the increased risk of severe allergic reactions, although rare, to chlorhexidine gluconate. The FDA advises healthcare professionals to thoroughly review the patient’s allergic history prior to using or prescribing chlorhexidine gluconate-based products [38]. At high doses, chlorhexidine can lead to toxic effects and damage, such as to the middle ear and cornea [37,39]. The range of allergic reactions can vary from mild skin symptoms to potentially life-threatening anaphylactic shock [40]. The hypersensitivity reactions that may occur can be type II (cytotoxic and immediate) or type IV (cell-mediated and delayed) [41]. In recent years, reports of allergic phenomena associated with chlorhexidine have significantly increased, particularly in the perioperative setting [42].

In the veterinary field, the use of antiseptic agents is inherently complex, as it requires the maintenance of high hygienic standards while simultaneously limiting the dissemination of antimicrobial-resistant pathogens naturally colonizing the skin microbiota [43,44]. In veterinary medicine, the challenges surrounding the use of antiseptics are magnified by the imperative to uphold stringent hygiene standards and mitigate the spread of resistant pathogens on skin that is naturally colonized by microbial flora [43,44]. This issue not only affects the health of animals but also extends to human and environmental health, given that antiseptics used in veterinary practices frequently overlap with those used in human healthcare. Of particular concern is the reduced susceptibility of certain pathogenic species to chlorhexidine, a widely used antiseptic, which may compromise its efficacy in clinical settings and contribute to the emergence of cross-resistance to other antimicrobial agents, such as colistin and daptomycin [45,46]. Furthermore, bacterial strains, including *Staphylococcus epidermidis*, *Staphylococcus aureus*, vancomycin-resistant enterococci (VRE), *Klebsiella pneumoniae*, and *Acinetobacter baumannii*, which exhibit resistance to multiple antibiotics, may harbor genetic determinants that elevate the minimum inhibitory concentration (MIC) of chlorhexidine, further complicating the management of infections [45,47].

In this context, veterinary medicine could greatly benefit from the adoption of protocols that integrate the use of ozone. Indeed, compared to other antiseptics, ozone has not been shown to present significant toxicity when used within safe dosages [28,29], making it preferable in cases of hypersensitivity to other antiseptics. Furthermore, several studies have highlighted its effectiveness not only in reducing microbial load but also in removing genes responsible for the spread of antibiotic resistance, known as antibiotic resistance genes (ARGs) [48]. This is particularly relevant because the reduction of these genes in the environment can limit the potential transfer of resistance between bacteria, helping to control the spread of the phenomenon. The bactericidal capacity of ozone against Gram-positive bacteria, Gram-negative bacteria, fungi, and spores is well established within the scientific community [17,19,31,32], particularly against resistant bacteria such as *Staphylococcus aureus*, *Pseudomonas aeruginosa*, and ESBL-producing *Klebsiella pneumoniae* [33,34], making it a valuable ally even for environmental and food antiseptic treatments [49,50].

Furthermore, the persistence of antimicrobial efficacy constitutes a pivotal determinant of surgical asepsis, directly influencing the maintenance of a sterile operative field and the prevention of bacterial recolonization during and after surgical interventions. Among currently available antiseptics, the prolonged bacteriostatic and bactericidal activity of chlorhexidine is extensively substantiated and represents a critical clinical advantage [36]. Conversely, the present study was deliberately confined to specifically investigate this rapid antimicrobial response; thus, the potential for sustained efficacy was not systematically evaluated. While this methodological decision inherently limits the scope of the findings, it underscores the preliminary framework of the investigation and provides a basis for future studies exploring approaches to enhance ozone’s residual activity or to examine its integration as an adjunctive modality within established antiseptic protocols.

## 5. Conclusions

This preliminary study demonstrates that the application of bidistilled ozone water for preoperative skin antisepsis in dogs achieves a bacterial load reduction comparable to that of chlorhexidine and ethyl alcohol. Given its favorable safety profile, rapid antimicrobial action, and minimal adverse effects at controlled concentrations, ozone represents a promising adjunct in veterinary surgical antisepsis protocols. While not intended to replace conventional antiseptics, the integration of ozone may enhance infection control strategies, particularly in the context of increasing antimicrobial resistance. Further rigorous investigations are warranted to optimize and fully elucidate the role of ozone in clinical practice.

## Figures and Tables

**Table 1:** Summary of antiseptic agents used and related sampling areas.

Primary Sampling Area	Antiseptic Treatment	First Sampling (90”)	Second Sampling (180”)
Control	-	-	-
Area 1 6 × 6 cm	Soap-based chlorhexidine + alcoholic chlorhexidine	Subzone 1 “*P1*” 3 × 6 cm	Subzone 1 “*S1*” 3 × 6 cm
Area 2 6 × 6 cm	Soap-based chlorhexidine + ozonated water (20 µg/mL)	Subzone 2 “*P2*” 3 × 6 cm	Subzone 2 “*S2*” 3 × 6 cm
Area 3 6 × 6 cm	Ethyl alcohol + ozonated water (20 µg/mL)	Subzone 3 “*P3*” 3 × 6 cm	Subzone 3 “*S3*” 3 × 6 cm
Area 4 6 × 6 cm	Ozonated water (20 µg/mL)	Subzone 4 “*P4*” 3 × 6 cm	Subzone 4 “*S4*” 3 × 6 cm

## Data Availability

The raw data supporting the conclusions of this article will be made available by the authors upon request.
